# Supplementary material for: Analysis of chi angle distributions in free amino acids via multiplet fitting of proton scalar couplings
Source: Magn Reson (Gott). 2024 Aug 19;5(2):103–20. doi: 10.5194/mr-5-103-2024 (PMC11570886; doi:10.5194/mr-5-103-2024)
Supplement: The supplement related to this article is available online at: https://doi.org/10.5194/mr-5-103-2024-supplement. [file mr-5-103-2024-supplement.zip › Syed2024/data/fit1d_gissmo.tar.gz!/fit1d/glu/500/bmse000037_simulation_1_nmredata.zip!/bmse000037_simulation_1_nmredata/1H/pdata/992/index.html_C=S_O=A]

Index of /ftp/pub/bmrb/metabolomics/standards/L\_glutamic\_acid/nmr/bmse000037/1H/pdata/992


# Index of /ftp/pub/bmrb/metabolomics/standards/L\_glutamic\_acid/nmr/bmse000037/1H/pdata/992

|  | Name | Last modified | Size | Description |
| --- | --- | --- | --- | --- |
| --- | | | | |
|  | Parent Directory |  | - |  |
|  | t1elim | 03-Oct-2011 11:05 | 60 |  |
|  | pt1t2.par | 03-Oct-2011 11:05 | 90 |  |
|  | dt1t2.txt | 03-Oct-2011 11:05 | 118 |  |
|  | t1\_disp | 03-Oct-2011 11:05 | 256 |  |
|  | t1ascii | 03-Oct-2011 11:05 | 412 |  |
|  | outd | 03-Oct-2011 11:05 | 505 |  |
|  | t1ints.txt | 03-Oct-2011 11:05 | 868 |  |
|  | t1peaks.txt | 03-Oct-2011 11:05 | 868 |  |
|  | t1par | 03-Oct-2011 11:05 | 1.0K |  |
|  | t1ints | 03-Oct-2011 11:05 | 1.4K |  |
|  | t1peaks | 03-Oct-2011 11:05 | 1.4K |  |
|  | t1t2.dx | 03-Oct-2011 11:05 | 1.5K |  |
|  | procs | 03-Oct-2011 11:05 | 1.7K |  |
|  | proc | 03-Oct-2011 11:05 | 1.8K |  |
|  | ct1t2.txt | 03-Oct-2011 11:05 | 2.3K |  |
| --- | | | | |

Apache/2.2.3 (CentOS) Server at www.bmrb.wisc.edu Port 80
